# Application of loop-mediated isothermal amplification assay in the detection of herpesvirus of turkey (FC 126 strain) from chicken samples in Nigeria

**DOI:** 10.14202/vetworld.2017.1383-1388

**Published:** 2017-11-26

**Authors:** A. J. Adedeji, P. A. Abdu, P. D. Luka, A. A. Owoade, T. M. Joannis

**Affiliations:** 1 Viral Research Division, National Veterinary Research Institute, Vom, Nigeria; 2 Department of Veterinary Medicine, Faculty of Veterinary Medicine, Ahmadu Bello University, Zaria, Nigeria; 3 Biotechnology Division, National Veterinary Research Institute, Vom, Nigeria; 4 Department of Veterinary Medicine, Faculty of Veterinary Medicine, University of Ibadan, Nigeria; 5 Regional Laboratory for Animal Influenza and Other Transboundary Animal Diseases, National Veterinary Research Institute, Vom, Nigeria

**Keywords:** herpesvirus of turkeys, loop-mediated isothermal amplification procedure, Nigeria

## Abstract

**Aim:**

This study was designed to optimize and apply the use of loop-mediated isothermal amplification (LAMP) as an alternative to conventional polymerase chain reaction (PCR) for the detection of herpesvirus of turkeys (HVT) (FC 126 strain) in vaccinated and non-vaccinated poultry in Nigeria.

**Materials and Methods:**

HVT positive control (vaccine) was used for optimization of LAMP using six primers that target the HVT070 gene sequence of the virus. These primers can differentiate HVT, a Marek’s disease virus (MDV) serotype 3 from MDV serotypes 1 and 2. Samples were collected from clinical cases of Marek’s disease (MD) in chickens, processed and subjected to LAMP and PCR.

**Results:**

LAMP assay for HVT was optimized. HVT was detected in 60% (3/5) and 100% (5/5) of the samples analyzed by PCR and LAMP, respectively. HVT was detected in the feathers, liver, skin, and spleen with average DNA purity of 3.05-4.52 μg DNA/mg (A260/A280) using LAMP. Conventional PCR detected HVT in two vaccinated and one unvaccinated chicken samples, while LAMP detected HVT in two vaccinated and three unvaccinated corresponding chicken samples. However, LAMP was a faster and simpler technique to carry out than PCR.

**Conclusion:**

LAMP assay for the detection of HVT was optimized. LAMP and PCR detected HVT in clinical samples collected. LAMP assay can be a very good alternative to PCR for detection of HVT and other viruses. This is the first report of the use of LAMP for the detection of viruses of veterinary importance in Nigeria. LAMP should be optimized as a diagnostic and research tool for investigation of poultry diseases such as MD in Nigeria.

## Introduction

Marek’s disease (MD) initially described by József Marek in 1907 is a highly contagious lymphoproliferative viral disease of poultry caused by MD virus (MDV) [1,2]. The MDV is a double-stranded linear DNA virus belonging to the family Herpesviridae, subfamily *Alphaherpesvirinae*, genus *Mardivirus* (GaHV-2) [3]. MDV is classified into three serotypes, *Gallidherpesvirus*-2 or serotype 1 (MDV-1), *Gallid herpesvirus* 3 or serotype 2 (MDV-2), and *Meleagrid herpesvirus* 1 (MDV-3, serotype 3), or herpesvirus of turkeys (HVT) [3]. Clinical MD is caused by MDV-1 strain, while MDV-2 and HVT are nonpathogenic and used as vaccine strains [2,4]. MD affects chickens, quails, turkeys, pheasants, and game fowl [3]. MDV is highly ubiquitous and contagious with long-term infectivity of poultry house environment and eradication of MD is very difficult [5]. Prevention of MD is, therefore, mainly based on vaccination, improved biosecurity, and selection for genetic resistance [5].

The development of MD vaccines was a major breakthrough in basic cancer research; moreover, it is the first neoplastic disease controlled by vaccination [6,7]. Despite the successes recorded with its usage, increase in virulence of MDV has accompanied the use of MD vaccines [7,8]. In addition, vaccination against MDV does not lead to a sterilizing immunity and vaccinated birds become protected from clinical disease but continue to shed and transmitted the virus [5,7].

Several advances in laboratory detection of MDV have been reported, particularly using molecular techniques such as polymerase chain reaction (PCR) and Real Time-PCR which can detect and quantify field and vaccine strains of MDV like HVT [9,10]. However, the requirement of using costly equipment for routine testing by small laboratories and field veterinarians remains a challenge [11]. Despite the availability of different detection techniques, there is still need for a rapid and simple molecular method that does not require expensive laboratory equipment. Alternative methods to standard PCR include nucleic acid sequence-based amplification (NASBA) and loop-mediated isothermal amplification (LAMP). LAMP is based on amplification of specific genetic loci [12]. LAMP also uses isothermal condition provided by a water bath or heating bloc and *Bst* or *Bsm* polymerases, which have DNA strand displacement activity, along with four to six primers [13]. LAMP technique can amplify a few copies of DNA to 10^9^ in <1 h under isothermal conditions [13].

The first report of MD in Nigeria was in 1962, subsequently several outbreaks have been reported in Nawathe *et al*. [14], Fatunmbi and Adene [15], Owoade and Oni [16], Wakawa *et al*. [17], Jwander *et al*. [18]. In a 2013 report, Nigeria was one of the countries with increasing prevalence of MD in the past 10 years, despite being one of the countries that routinely vaccinates chickens against MD [19]. In spite of routine vaccination, poultry farmers in Jos, Plateau State have been reporting outbreaks suspected to be MD.

This study was designed to optimize and apply the use of LAMP as an alternative to PCR for the detection of HVT in vaccinated and unvaccinated poultry in Nigeria.

## Materials and Methods

### Ethical approval

Ethical approval was not considered, because clinical samples were collected from dead chickens submitted for routine diagnosis at the Veterinary Clinic.

### Sample collection

Samples were collected from clinical cases of MD presented to a Veterinary Clinic in Jos, Plateau State, Nigeria. The samples were collected in 2014 and transported on ice and stored at -70°C in the Viral Research Division of the National Veterinary Research Institute, Vom, Plateau State, Nigeria. The vaccination history and postmortem findings of each case were recorded.

### DNA extraction

One gram of tissue from each sample was weighed and homogenized using pestle and mortar with sterile glass. Thereafter, 9 ml of phosphate-buffered saline was added and centrifuged in a refrigerated centrifuge at 10,000 rpm for 5 min to make 10% tissue suspension. The supernatant was decanted into a sterile tube and kept at 4°C for DNA extraction and the pellet discarded into a disinfectant. The viral DNA extraction was carried out using QIAamp DNA Mini kit from Qiagen (Qiagen, Hilden, Germany) following the manufacturer’s instructions. DNA extracts were evaluated by spectrophotometry (BioPhotometer; Eppendorf Scientific, Hamburg, Germany) at A260/A280 and checking on 1.5% agarose gel electrophoresis and kept at +4°C before use.

### Primers for PCR and LAMP

The published PCR and LAMP primer sequences used for this study are listed in Table-1 [20]. The primers were designed based on the HVT070 gene sequence of HVT (FC-126 Strain) with accession number NC_002641.1 which can differentiate MDV-3 or HVT from MDV-1 and MDV-2. The outer primers, MDV-3, F3 and MDV-3 B3, were used for the PCR and all for LAMP. The LAMP primers consist of three set of primers, the outer primers MDV-3 F3 and B3, inners primers (MDV-3 FIP and BIP), and additionally loop primers (MDV-3 LF and MDV-3 LB) designed to accelerate the LAMP reaction [20].

**Table-1:** The list of primer sequences of HVT (FC-126) used for polymerase chain reaction and loop-mediated isothermal amplification procedure.

Primer ID	Sequence (5’–3’)	Marek’s disease virus serotype	Reference
MDV-3F3	ATAAATTATATCGCTAGGACAGAC	HVT (FC 126 strain)	Woźniakowski *et al*. [20]
MDV-3B3	ACGATGTGCTGTCGTCTA		
MDV-3FIP	CCAGGGTATGCATATTCCATAACAGTTTTCCAAACGACCTTTATCCCA		
MDV-3BIP	CCAGAAATTGCACGCACGAGTTTTAGAATTTGTGCATTTAGCCTT		
MDV-3LF	TTGAGAAGAGGATCTGACTG		
MDV-3LB	GCGTCATTGGTTTTACATTT		

HVT=Herpesvirus of turkeys, MDV=Marek’s disease virus

### Optimization of LAMP

LAMP assay was optimized using different concentrations of all the primers and reagents in the kit (OmniAmp™ RNA and DNALAMP Kit, Lucigen USA). These were conducted as follows: Betaine (0.2, 0.3, 0.4, 0.5M), OmniAmp DNA Polymerase 50× (0.5, 1 µl), MgSO_4_ (0, 4,8, 12 mM), 10 mM dNTPs (0.8, 1.6, 2, 4 µl), DNA template (1.5, 2, 2.5 µl of average 3.05-4.52 μg DNA/mg), outer primers F3 and B3 (7.5, 10 pmol), inner primers FIP and BIP (40, 60 pmol), loop primers LF and BF (15, 20 pmol), different temperature reaction conditions (60, 65, 68, 70°C), and time (25, 30, 35 min) as well as termination temperature (80, 95°C) and time (2, 5 min).

### LAMP

The LAMP mix (OmniAmp™ RNA and DNA LAMP Kit, Lucigen, Middleton WI, USA) was done at two different times in 25 µl volume mix consisting of 10X DNA polymerase buffer C 2.5 µl, OmniAmp DNA polymerase 50× 1 µl, the primers were MDV F3 and B3 10 pmol each, MDV LF and LB 20 pmol each, MDV FIP and BIP 40 pmol each, 100 mM MgSO_4_ 4 mM, dNTPs 4 µl, Betaine 0.4M, and nuclease-free water and DNA template 2 µl. The LAMP amplification reactions were performed at 70°C for 30 min and subsequently enzyme activity was stopped by holding the mix on ice at 4°C while a second stage incubation was done at 95°C for 5 min. The amplified products were analyzed on a 1.5% agarose gel and visualized by staining with ethidium bromide. LAMP-positive samples showed specific ladder-like products ranging from 197 to 1300 bp. Bands below the 197-bp product were considered to be primer-dimers.

### PCR

PCR was carried out in 25 µl volume reaction mix consisting of Thermo Scientific Dream Taq Green PCR Master Mix (2×) 12.5 µl, 20 pmol of MDV F3 and B3 primers, nuclease-free water and DNA, 2 µl. The thermal cycling profile included initial denaturation 94°C for 3 min followed by 35 cycles at 94°C for 30 s, 55°C for 30 s, and 72°C for 1 min for denaturation, annealing, and extension, respectively, and final extension at 72°C for 7 min. HVT (FC-126) vaccine (ABIC, Israel) was used as positive control and aliquot of molecular grade water was used as the negative control and were both subjected to DNA extraction and PCR. The expected band size of positive samples is 200 bps. GelPilot DNA molecular Weight Marker (QIAGEN) 100 bps was used to run the samples.

## Results

### Optimized LAMP

**Figure-1:**
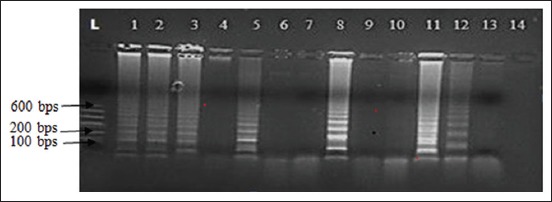
Agarose gel of loop-mediated isothermal amplification (LAMP) with product of herpesvirus of turkeys DNA positive control using the LAMP primers. Lanes 1-14 were the results using different concentration of reagents. Lanes 1-3 (positive control in the kit), Lane 4 (10× buffer C 2.5 µl, OmniAmp DNA Polymerase 50× 0.5 µl, 100 mM MgSO_4_ 0 mM, dNTPs 0.8 µl, Betaine 0.2 M, and DNA template 1.5 µl) Lane 5 (10× buffer C 2.5 µl, OmniAmp DNA Polymerase 50× 1 µl, 100 mM MgSO4 4 mM, dNTPs 1.6 µl, Betaine 0.3 M, and DNA template 1.5 µl) Lanes 6-7 (10× buffer C 2.5 µl, OmniAmp DNA Polymerase 50× 0.5 µl, 100 mM MgSO_4_ 1.6 mM, dNTPs 2 µl, Betaine 0.3 M, and DNA template 1.5 µl), Lane 8 (10× Buffer C 2.5 µl, OmniAmp DNA Polymerase 50× 1 µl, 100 mM MgSO_4_ 4 mM, dNTPs 4 µl, Betaine 0.4 M, and DNA 2 µl). Lanes 9-10 (10× buffer C 2.5 µl, OmniAmp DNA Polymerase 50× 1 µl, 100 mM MgSO_4_ 8 mM, dNTPs 1.6 µl, Betaine 0.4 M, and DNA 2.5 µl). Lanes 11 (10× buffer C 2.5 µl, OmniAmp DNA Polymerase 50× 1 µl, 100 mM MgSO_4_, 4 mM dNTPs 4 µl, Betaine 0.4 M, and DNA 2.5 µl). Lane 12 (10× buffer C 2.5 µl, OmniAmp DNA Polymerase 50× 1 µl, 100 mM MgSO4 4 mM, dNTPs 4 µl, Betaine 0.4 M, and DNA 1 µl). Lane 13 (10× buffer C 2.5 µl, OmniAmp DNA Polymerase 50× 1 µl, 100 mM MgSO4, 8 mM dNTPs 1.6 µl, Betaine 0.4 M, and DNA 2 µl). Lane 14 (10× buffer C 2.5 µl, OmniAmp DNA Polymerase 50× 1 µl, 100 mM MgSO4, 12 mM dNTPs 2 µl, Betaine 0.4 M, and DNA 1.5 µl).

**Figure-2:**
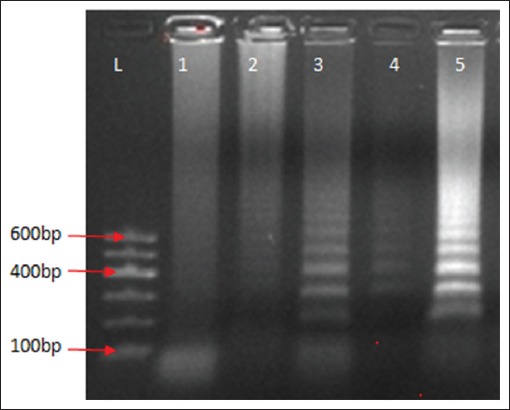
Agarose gel of loop-mediated isothermal amplification procedure DNA product of herpesvirus of turkeys at different temperature conditions; Lane 1 (60°C), Lane 2 (65°C), Lane 3-4 (68°C), Lane 5 (70°C). L is 100 bps DNA marker (GelPilot, QIAGEN).

**Figure-3:**
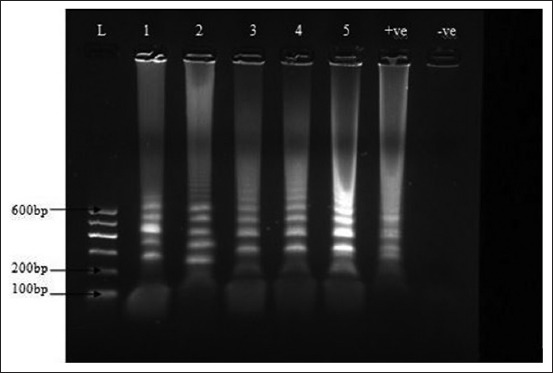
Loop-mediated isothermal amplification (LAMP) procedure DNA product using herpesvirus of turkeys LAMP specific primers, −ve was the negative control, +ve was the positive control, 2-5 were positive. 1 was spleen of broiler, 2 was liver sample of broiler, 3 and 4 feather of layer 5 was DNA skin of indigenous local chicken. L is 100 bps DNA marker (GelPilot, QIAGEN).

Based on the different variables adjusted, LAMP mix was carried out as described in the materials and methods. The incubation protocol and the LAMP mix with the bright and distinct band patterns of different sizes were subsequently used to run LAMP on clinical samples collected from the field (Figures-1 and 2). The LAMP mix with the bright and distinct band patterns of different sizes were 10× DNA Polymerase Buffer C 2.5 µl, OmniAmp DNA Polymerase 50× 1 µl, the outer primers were 10 pmol each, loop primers 20 pmol each, inner primers 40 pmol each, 100 mM MgSO_4_ 4 mM, dNTPs 4 µl, Betaine 0.4M, and DNA 2 µl (Figure-3). The optimized LAMP incubation protocol was initial incubation temperature of 70°C for 30 min and termination temperature of 95°C for 5 min. HVT (FC-126) vaccine (ABIC, Israel) was used as positive control, and nuclease-free water was used as the negative control and were both subjected to DNA extraction and LAMP.

### Detection of HVT in clinical samples of chickens by PCR and LAMP

**Figure-4:**
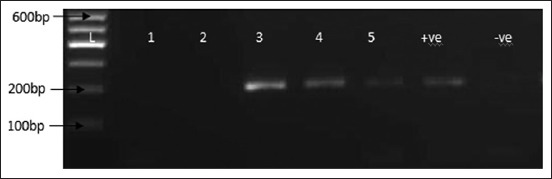
Agarose gel of polymerase chain reaction amplification product of herpesvirus of turkeys FC 126 using specific primers, the positive band was at 200 bps +ve was the positive control, −ve control. L is 100 bps DNA marker (GelPilot, QIAGEN), lanes 1-2 were negative samples and lanes 3-5 were positive samples.

Samples collected were from broilers (2), layers (2) and indigenous free-range chicken (1), the samples were spleen (1), liver (1), and feathers (2), and skin (1) (Table-2 and Figure-3) [20]. The postmortem findings included splenomegaly, hepatomegaly, and skin tumors as listed in Table-2. The layers were vaccinated using HVT by the farmers, while the broilers and the local chicken had no vaccination history. Of the samples collected 60% (3/5) and 100% (5/5) were positive for HVT by PCR (Figure-4) and LAMP (Figure-3), respectively. Moreover, HVT was detected in the spleen, feathers, and liver. PCR detected HVT in two of the flocks that were vaccinated and the indigenous local chicken, while HVT was detected in both vaccinated and unvaccinated flocks by LAMP (Table-2). On repetition of the procedure on a different day and on the same set of samples yielded similar results.

**Table-2:** Postmortem findings and results detection of HVT in clinical samples from poultry by PCR and LAMP in Jos, Plateau State, Nigeria.

Type of chicken	Postmortem findings	Age in weeks	Vaccination history	Type of sample	PCR results	LAMP results
Broiler	Splenomegaly	9	Not vaccinated against MD with HVT	Spleen	-	+
Broiler	Splenomegaly, hepatomegaly, skin tumors	10	Not vaccinated against MD with HVT	Liver	-	+
Layer	Hepatomegaly splenomegaly and enlarged kidneys and emaciation	60	Vaccinated twice with HVT before day 21 of age	Feather	+	+
Layer	Hepatomegaly, splenomegaly	40	Vaccinated with HVT	Feather	+	+
Local indigenous chicken	Hepatomegaly splenomegaly and skin tumors	Unknown	Unknown	Skin	+	+
Total					3/5	5/5

HVT=Herpesvirus of turkeys, MD=Marek’s disease, PCR=Polymerase chain reaction, LAMP=Loop-mediated isothermal amplification

## Discussion

LAMP has been used for the detection of viruses of veterinary importance in several countries as a rapid diagnostic tool [21-26]. The use of LAMP as a tool for the detection of viruses affecting livestock has not been reported in Nigeria. The results obtained in this study revealed the optimization of LAMP protocol for HVT (FC-126 strain) and HVT detected in 100% of the clinical samples collected compared to 60% detection by PCR in the same samples. In addition, LAMP technique was simpler and results turnover time was faster compared to PCR. Although our aim was not to compare the two protocols our findings are in agreement with several studies in which LAMP was reported to be more sensitive than PCR [11,25,27]. A major challenge for field veterinarians in Nigeria is lack of laboratory support in the investigation of MD and other livestock diseases. Development and adoption of LAMP for pen-site rapid diagnosis of MD and other poultry diseases will strengthen the confidence of veterinarians on the quality of veterinary service delivery and control of the spread of livestock diseases. The use of LAMP can also enhance research on poultry diseases in Nigeria. Vaccination against MD is routinely done in Nigeria using HVT (FC-126 strain) and LAMP can also be used for field detection and monitoring to ensure effective vaccination against MD [20,28]. Moreover, in recent years, HVT is being widely used as a vector of other poultry viruses for the production of vaccines [29-32]. Hence, LAMP may be used for field monitoring of these vaccines. A very important finding in this study is the detection of HVT in unvaccinated broilers and indigenous local chickens diagnosed to be having MD based on clinical and gross pathological findings. Although the vaccination status of the indigenous local chicken is unknown, most indigenous local chickens in Nigeria are usually not vaccinated and kept on free range by rural farmers. HVT like most MDV serotypes is transmitted horizontally and hence the broilers and indigenous chicken may have been infected from highly contaminated environment. Studies have revealed that MDV vaccines strains are shed into the environment alongside the pathogenic MDV and transmitted effectively between chickens [4,33]. This could be the scenario in this study in which HVT was detected in unvaccinated broilers and local chicken. Although MDV-2 and HVT are not pathogenic, these viruses infect poultry just like all MDV with livelong infectivity which implies that they are continuously shed into the environment through feather follicles [34].

## Conclusion and Recommendations

LAMP assay for the detection of HVT was optimized and used to detect HVT in clinical samples collected from chickens. LAMP assay can be a very good alternative to PCR for the detection of HVT and other viruses. This is the first report of the use LAMP in detection of a virus of veterinary importance in Nigeria. Based on this study, it is recommended that LAMP be optimized as a diagnostic and research tool for viral diseases of poultry such as MD in Nigeria.

## Authors’ Contributions

AJA, PAA, and OAA designed the work, AJA collected the samples, AJA and LPD carried out the laboratory analysis. AJA, PAA, TMJ, and LPD wrote the manuscript, TJM and AJA provided the reagents and materials. All authors read and approved the final manuscript.
